# Advancing the understanding of research during medical education through collaborative learning: the Collaboration of Practitioners and Researchers Seminar Series

**DOI:** 10.1186/s12909-019-1890-6

**Published:** 2019-12-10

**Authors:** Charles Yin, Alexander J. Moszcyznski, Jessica N. Blom, Tristan P. E. Johnson, Douglas L. Jones

**Affiliations:** 1 Schulich School of Medicine and Dentistry, Western University, London, ON Canada; 2 Department of History, Western University, London, ON Canada

**Keywords:** Translational research, Collaborative learning, Student-led, Medical student, Graduate student

## Abstract

**Background:**

The Collaboration of Practitioners and Researchers Seminar Series is student-led program comprised of seminars delivered jointly by medical and graduate students on a topic in medicine of mutual interest to an audience of both medical and graduate students.

**Methods:**

Following its inaugural year in 2016–2017, we evaluated changes in attendees’ perceived understanding of translational research through an electronic survey and semi-structured interviews with attendees.

**Results:**

Study participants rated their understanding of translational research and comfort with interacting with students from the other program higher following attending seminars. Participants believed that the seminars helped in breaking barriers between medical and graduate students.

**Conclusions:**

We conclude that this seminar series positively impacted attendees’ understanding of translational research and attitudes towards collaboration between medical and graduate students. We believe that similar initiatives may be of value in fostering new opportunities for collaboration between medical and graduate students at other institutions.

## Background

Since the early 1990s, evidence-based medicine has taken an increasingly important role in medical practice [1–3]. Medical schools must demonstrate commitment to research by “provid[ing] sufficient opportunities, encouragement, and support for medical student participation in research” [4]. However, there is often little formal research teaching at most medical schools [5–8]. As a result, some medical students intending to pursue research-intensive careers lose interest in research during medical school [9]. While not every medical student will pursue a research-intensive career, physicians should possess the capacity to understand the scientific literature and critically interpret research findings [10–12]. Biomedical research increasingly involves collaboration between physicians and non-clinical colleagues, which improves research impact [13]. Although interprofessional education between healthcare practitioners is a familiar concept, interprofessional collaboration between medical and graduate students remains rare and tends to be viewed negatively by both groups [14].

To create an avenue for collaboration between medical and graduate students early in their training, we created—to the best of our knowledge—the first *student-led* seminar series featuring collaborative delivery of content involving both student groups. Entitled the “Collaboration of Practitioners and Researchers Seminar Series” (CPRSS), this series featured bi-monthly seminars that were prepared and delivered by a group of both medical and graduate students on biomedical topics. Medical students provided the clinical perspective, while graduate students discussed ongoing research in the field, often including their own work. To evaluate whether CPRSS led to an improved collaborative environment between medical and graduate students, we employed an electronic survey and semi-structured interviews to explore attendees’ perceived understanding of translational research and attitudes towards collaboration with the other group.

## Methods

### Research participants and ethics

Participants for this study were recruited from medical and graduate students at the Schulich School of Medicine and Dentistry (London, Canada) who attended CPRSS seminars during 2016–2017. This study was reviewed and approved by the Office of Human Research Ethics at Western University Non-Medical Research Ethics Board (NMREB Reference Number: 109484).

### Electronic survey

All participants completed a survey that retrospectively assessed their perceptions around translational research and the impact of attending CPRSS using Likert-like scales. Statistical significance was assessed using the Wilcoxon matched-pairs signed rank test with Pratt’s method. Analysis was performed using GraphPad Prism 6 (GraphPad, California).

### Participant interviews

Survey participants were invited to participate in a semi-structured interview assessing experiences with CPRSS. Interviews were conducted by one investigator (TPEJ) with identities of the interviewees being blinded to the other investigators. Transcripts were analyzed by three investigators who did not participate in the interview. Open codes were assigned to each line of the transcripts and subsequently sub-themes and themes were identified. Trustworthiness of this qualitative analysis was ensured by multiple means. First, interview data were gathered from both medical and graduate student attendees. Interview transcripts were independently analyzed by three investigators. The setting of CPRSS is described previously and care was taken by the investigators to collect information regarding the background of each participant.

## Results

### Electronic survey

**Fig. 1:**
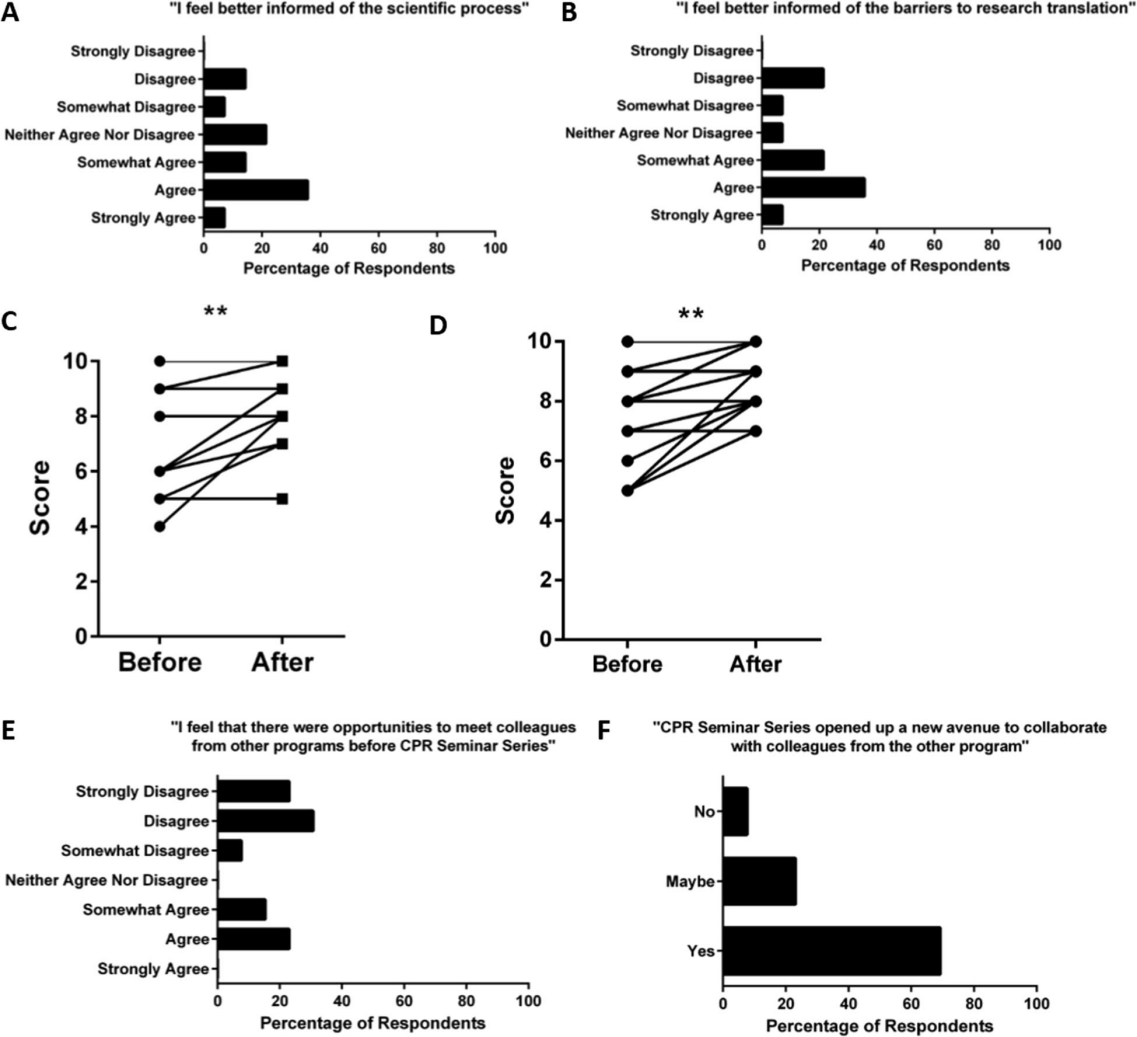
Involvement in the Collaboration of Practitioners and Researchers Seminar Series increases students’ understanding of the scientific process and translational research. Survey participants were asked to indicate the degree to which they agreed or disagreed with a series of statements examining their self-perceived understanding of the scientific process (**a**), barriers to translational research (**b**). Participants were then asked to rank their self-perceived understanding of translational research (**c**) and likelihood of pursuing a career involving translational research (**d**) on a 10-point Likert-like scale before and after involvement with the Collaboration of Practitioners and Researchers Seminar Series. Participants were also asked to indicate their agreement with a series of statements exploring whether there are sufficient existing opportunities to interact with colleagues from the other program (**e**) or whether the seminars represented a new opportunity for collaboration (**f**). **p < 0.01 by Wilcoxon matched-pairs signed rank test with Pratt’s correction

Of the 93 students who attended CPRSS in 2016–2017, 14 students (response rate: 15%) completed the survey. A majority felt they were better informed about the scientific process and barriers to research translation following seminar attendance (Fig. 1a, b). Perception of understanding of translational research was significantly increased post-seminar attendance as compared to pre-seminar (*p* = 0.004, Fig. 1c). Students also indicated that they were significantly more interested in pursuing a career in translational research (*p* = 0.008, Fig. 1d). A total of 9/14 students surveyed (64%) felt that there were insufficient opportunities to meet colleagues from other programs prior to CPRSS (Fig. 1e) and 10/14 (71%) agreed that CPRSS opened a new avenue to collaborate with students from the other program (Fig. 1f).

### Participant interviews

Semi-structured interviews were conducted with two medical students (**M1** and **M2**) and two graduate students (**G1** and **G2**). One medical student (**M1**) had taken undergraduate epidemiology courses while the other (**M2**) held a graduate degree in biochemistry. Transcripts were analyzed using an inductive approach and emergent themes were identified through an iterative process of discussion between investigators (Table 1). Major themes identified were: breaking barriers, building skills, and educational value.

**Table 1:** Emergent themes and sub-themes

	Sub-themes	Themes
Investigator 1	Breaking barriers	Breaking barriers
	Lack of exposure/opportunity	
	Practice catering to audience	
	Building confidence/risk taking	
	Missing skill sets	
	Collegiality	
Investigator 2	Positive experience	Building skills
	Barriers	
	Understanding/lack of understanding	
	Intergroup collaboration	
	Extra-educational value	
	Skill building	
	Segregation	
Investigator 3	General positive experience	
	Collaboration (good/bad)	Educational value
	Student-led nature	
	Skill development	
	Career preparation	
	Lack of research training	
	Sharing research	
	Clinical translation	

Students from both groups felt that they experienced *institutional barriers* in their current education including a *lack of opportunity* and *segregation*. Both medical and graduate students experienced a *lack of opportunity* to involve themselves in collaboration with the other group. Interestingly, this lack of opportunity was partially attributed to a sense of *segregation* from one another: “I feel like the medical student world and the graduate student world they’re two very separate worlds. So we don’t often get to interact with them” (**G2**). Students felt that CPRSS contributed to an increase in *collegiality* and *collaboration* between groups and as such represented a unique opportunity to enhance their education.

Both groups of students felt that participation in CPRSS led to the development of skills that would benefit them in their careers. Increasing the amount of c*ommunication* with one another was valued by both student groups: “I want to share this research and also I wanted to hear about what treatments are available now in the clinical field” (**G1)**. Medical students especially felt that this was an avenue for *career development* via extra research exposure: “A lot of us medical students need or want to do research in order to … advance our career prospects” (**M1**).

Students felt that the *informal setting* contributed to an increased willingness to participate, thus leading to an increase in educational value. Both graduate and medical students felt that the *informal setting* led to a greater opportunity to be creative and be more open in communication. This led to a better learning experience: “It doesn’t make you … shy away or be afraid to … speak out about your opinions and your thoughts without having some sort of fear being shut down by some senior person.” (**G2**).

## Discussion

Teaching on research and scholarship is a necessary part of medical education. In this study, we report trainee perceptions of translational research understanding following participation in a student-led translational research seminar at a single Canadian institution. Participation in the seminars led to an increase in students’ assessment of their own understanding of translational research and in their interest in pursuing a career involving translational research. The effectiveness of CPRSS was attributed to its ability to break down barriers between medical and graduate students, provide an opportunity to build skills, and provide mutually-beneficial educational value.

There are several important limitations to this study. First, a limited sample size with just 14 survey respondents limits the generalizability of our findings, which we attempted to address through employing interviews to further enrich our data. Another limitation is the measurement of perceived rather than real changes in understanding translational research and attitudes towards collaboration. These limitations highlight the need for a future study with a larger sample size and objective measures.

## Conclusion

We conclude that, despite several limitations, the present data represent a first step in this direction and will allow other institutions to consider and design similar programs that will increase collaboration between medical and graduate students.

## Data Availability

The datasets used and/or analysed during the current study are unsuitable for deposition in a repository due to their limited nature, and instead are available from the corresponding author on reasonable request.

## Abbreviations

CPRSS: Collaboration of Practitioners and Researchers Seminar Series
NMREB: Non-Medical Research Ethics Board
